# Association between an angiotensin-converting enzyme gene polymorphism and Alzheimer’s disease in a Tunisian population

**DOI:** 10.1186/s12991-017-0164-0

**Published:** 2017-11-17

**Authors:** Najiba Fekih-Mrissa, Ines Bedoui, Aycha Sayeh, Hajer Derbali, Meriem Mrad, Ridha Mrissa, Brahim Nsiri

**Affiliations:** 1 Laboratory of Molecular Biology, Department of Hematology, Military Hospital of Tunisia, Mont Fleury, 1008 Tunis, Tunisia; 2 Department of Neurology, Military Hospital of Tunisia, Montfleury, Tunis, 1008 Tunisia

**Keywords:** Angiotensin-converting enzyme gene, Alzheimer’s disease

## Abstract

**Background:**

The angiotensin-converting enzyme gene (ACE) insertion/deletion (I/D or indel) polymorphism has long been linked to Alzheimer’s disease (AD), but the interpretation of established data remains controversial. The aim of this study was to determine whether the angiotensin-converting enzyme is associated with the risk of Alzheimer’s disease in Tunisian patients.

**Methods:**

We analyzed the genotype and allele frequency distribution of the ACE I/D gene polymorphism in 60 Tunisian AD patients and 120 healthy controls.

**Results:**

There is a significantly increased risk of AD in carriers of the D/D genotype (51.67% in patients vs. 31.67% in controls; *p* = .008, OR = 2.32). The D allele was also more frequently found in patients compared with controls (71.67% vs. 56.25%; *p* = .003, OR = 2.0). Moreover, as assessed by the Mini-Mental State Examination, patient D/D carriers were more frequently found to score in the severe category of dementia (65%) as compared to the moderate category (32%) or mild category (3%).

**Conclusions:**

The D/D genotype and D allele of the ACE I/D polymorphism were associated with an increased risk in the development of AD in a Tunisian population. Furthermore, at the time of patient evaluation (average age 75 years), patients suffering with severe dementia were found predominantly in D/D carriers and, conversely, the D/D genotype and D allele were more frequently found in AD patients with severe dementia. These preliminary exploratory results should be confirmed in larger studies and further work is required to explore and interpret possible alternative findings in diverse populations.

## Background

Alzheimer’s disease (AD) is a common cause of morbidity and mortality among the elderly and is characterized by progressive memory loss and cognitive dysfunction [1]. The brains of AD patients are essentially characterized by neuronal and synaptic loss, extracellular plaques composed of amyloid-β peptides, and intra-neuronal neurofibrillary tangles. Senile plaques composed mainly of amyloid-β (Aβ) are particularly important in the pathology of AD [2]. Among the Aβ-related genes, angiotensin-converting enzyme gene (ACE) is closely related to the production and degradation of Aβ [3]. However, ACE also catalyzes the formation of the vasoconstrictor angiotensin II (Ang II) from angiotensin I. The actions of Ang II within the central nervous system are also of increasing interest in the context of Alzheimer’s disease (AD). Ang II inhibits the release of acetylcholine (ACh) and has a pro-inflammatory effect [4].

The ACE gene is located on chromosome 17q23. The most common polymorphism of the ACE gene is the insertion/deletion (I/D) variant of 287 base pairs in intron 16. This polymorphism has been suggested to be associated with serum ACE protein levels [5], the specific activity of the ACE protein domain [6], and the transcriptional activity of the ACE gene promoter region [7]. Furthermore, ACE may lower amyloid-β levels by promoting its degradation and, thereby, reinforce the hypothesis of the role of ACE in the pathogenesis of AD [8]. However, a more nuanced hypothesis supposes divergent roles of ACE in the degradation of Aβ. ACE may first mediate a short-term neuroprotective action but the production of Ang II then may lead to longer term and more wide-ranging deleterious consequences (i.e., hypertension, damage to the blood–brain barrier, reduced cerebral blood flow, Aβ deposition, inflammation, and reduced cholinergic activity) [4]. Therefore, in either explanation of pathogenesis, the ACE I/D polymorphism becomes an important consideration as a risk factor for AD susceptibility. Perhaps because of these diverse actions of ACE, potential associations with AD have been examined in a great number of studies worldwide but have generated equivocal results [9–11].

This current study examined the genetic polymorphism (I/D) of the ACE gene in a group of AD individuals and controls. The purpose was to determine the nature of the relationship between the ACE gene and the risk of AD in Tunisian subjects.

## Methods

### Study population

We studied 60 patients with AD (20 female, 40 male) recruited from the Department of Neurology at the Military Hospital of Tunis. The mean age was 75.18 years ± 5.31 (SD). The diagnoses of probable AD were based upon the National Institute of Neurological and Communicative Diseases and Stroke/Alzheimer’s Disease and Related Disorders Association (NINCDS–ADRDA) clinical diagnostic criteria and Diagnostic and Statistical Manual of Mental Disorders (DSM IV) criteria with no clinical or laboratory evidence of a cause other than AD for dementia [12, 13]. All participants underwent a complete clinical investigation that included medical history, neurological and neuropsychological examinations (Mini-Mental State Examination (MMSE) [14], clock-drawing tests, the 5-word test, auditory verbal learning test, and Frontal Assessment Battery), screening laboratory tests, and neuro-imaging consisting of CT-scan and/or magnetic resonance imaging (MRI). MRI scans displayed substantial reduction in the volume of the medial temporal lobe and hippocampus in patients with Alzheimer’s disease as compared to controls.

Additionally, the control group consisted of 120 age- and gender-matched subjects (46 female, 74 male) with diverse Tunisian origin similar to that of the patients and chosen based on their medical history and physical examination. Their cognitive function was assessed using the MMSE examination [14] with all scores exceeding 26. Further, the controls did not exhibit any signs of dementia and reported no family history of AD or dementia.

All participants (or their guardians) in this study gave their fully informed consent. The study protocol was approved by the Ethics Committee of the Military Hospital of Tunisia and has therefore been performed in accordance with the ethical standards laid down in the 1964 Declaration of Helsinki and its later amendments.

### Laboratory methods

**Fig. 1:**
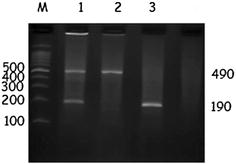
Agarose gel electrophoresis of PCR amplified products of the ACE (I/D) gene polymorphism. M represents 100-bp DNA ladder; lane 3 as DD genotype (190 bp); lane 2, as II genotype (490 bp) and lane 1, as ID genotypes (490 and 190 bp) of the ACE gene

Genomic DNA was extracted from peripheral blood leukocytes with a DNA extraction kit (QIAmp blood kit, Qiagen GmbH [Hilden, Germany]) according to the manufacturer’s protocol. ACE genotyping was carried out by polymerase chain reaction using oligonucleotide sense primer 5′-CTG GAG ACC ACT CCC ATC CTT TCT-3′, and the antisense primer 5′-GAT GTG GCC ATC ACA TTC GTC AGA T-3′. DNA samples (100 ng) were subjected to 35 cycles of PCR amplification under the following conditions: initial denaturation at 94 °C for 5 min (min), denaturation at 94 °C for 45 s (s), annealing at 58 °C for 1 min, extension at 72 °C for 45 s, and final extension at 72 °C for 7 min. The PCR products were electrophoresed on a 2% agarose gel stained with ethidium bromide to visualize three patterns: I/I (a 490-bp fragment), D/D (a 190-bp fragment), and I/D (both 490- and 190-bp fragments) (Fig. 1).

### Statistical analysis

All analyses related to the case–control study were performed using the Statistical Package for the Social Sciences v.16 (IBM, Armonk, NY USA). Data on quantitative characteristics are expressed as mean ± SD. Differences between cases and controls were evaluated by using the Chi-square test or Fisher’s exact test for qualitative variables. The Chi-square goodness-of-fit test was used to assess the genotype and allele frequencies for deviations from Hardy–Weinberg equilibrium. The Chi-square test of independence was used to ascertain possible dependences between MMSE scores of patients and genotypes. In addition, the odds ratio (OR) and 95% confidence intervals (CI) were calculated with two-by-two contingency table methods to measure the strength of associations (effect sizes). The ACE gene I/D polymorphism and the clinical characteristics of patients were compared to controls using Chi-square or Fisher exact tests (when cell frequencies were small). Probability values (*p* values) less than .05 were considered statistically significant.

## Results

The demographic data for all subjects are presented in Table 1. The mean ages (± SD) of the cases and controls were 75.18 years (± 5.31) and 72.94 years ± (5.0). There were no significant differences in any of the mean values regarding blood chemistry, including triglycerides and cholesterol, between the AD and control groups. MMSE scores, however, differed significantly between patients with AD and controls (*p* < .001). Both the case and control groups were in Hardy–Weinberg equilibrium (*χ* ^2^ = .014, *p* = .90; *χ* ^2^ < 10^−3^, *p* = .99; respectively). The ACE gene I/D genotype distributions and allele frequencies of the study groups are presented in Table 2. The analysis of genotype frequencies revealed an over-representation of the D/D genotype among patients compared to that of the control group (51.67% vs. 31.67%, respectively). This observation indicates an increased susceptibility of the D/D genotype carriers to AD (*p* = .008, OR = 2.32). However, the difference of the I/D genotype frequencies between patients and controls was not significant (*p* = .24). The I/I genotype was observed among 5 patients (8.33%) and 23 controls (19.17%) (*p* = .06). The ACE allele frequencies revealed that the D allele was found to be predominant among the AD group (71.67%) while the I allele was predominant among the control subjects (43.75%) with a statistically significant difference (*p* = .003, OR = 2.0). The D allele is statistically associated with an increased risk of AD in our population.

**Table 1:** Comparison of clinical variables in Alzheimer patients and controls

Clinical variable	Patients (*N* = 60) *n* (%)	Controls (*N* = 120) *n* (%)	*p* value
Gender F/M	20 (33.3)/40 (66.6)	46 (38.3)/74 (61.6)	–
Age years ± SD	75.18 ± 5.31	72.94 ± 5.0	–
Age of onset years ± SD	69 ± 4.48	–	–
MMSE	14 (6–22)	28 (> 26)	*<* *10* ^*−3*^
Tobacco use	12 (20)	30 (25)	.45
Diabetes	13 (21.6)	18 (15)	.26
Hypertension	15 (25)	20 (16.6)	.18

Significant *p* values in italics

**Table 2:** Genotype and allele frequencies of the ACE polymorphism in AD patients and controls

ACE I/D	Patients *N* = 60	Controls *N* = 120	*χ* ^2^	OR [CI 95%]	*p* value
	*n* (%)	*n* (%)			
II	5 (8.33)	23 (19.17)	3.57	.38 (.13–1.06)	.06
ID	24 (40.00)	59 (49.17)	1.35	.69 (.37–1.28)	.24
DD	31 (51.67)	38 (31.67)	6.7	2.32 (1.21–4.34)	*.008*
I	34 (28.33)	105 (43.75)	8.02	.5 (.30–.80)	*.003*
D	86 (71.67)	135 (56.25)		2 (1.25–3.33)	

*CI* confidence interval, *OR* odds ratio, *p value* probability value

Significant *p* values are in italics

In an effort to discern any relationship between genotypes (alleles) and the severity of dementia, Table 3 details the allocation of patient genotypes and alleles among three categories of Mini-Mental State Examination (MMSE) scores. The Chi-square test of independence broadly indicates that these MMSE categories are dependent upon genotypes ($\chi_{df\, = \,\,4}^{2}$ = 18.1, *p* = .001) and alleles ($\chi_{df\, = \,\,2}^{2}$ = 18.1, *p* < 10^−3^). In particular, the D/D genotype appears in 65% of patients with severe dementia, in 32% with moderate dementia, and in only 3% of those with mild dementia (as measured by the MMSE). Conversely, there are 26 patients with severe dementia and, of them, 77% are D/D carriers, 23% are I/D carriers, and there are no I/I carriers. Although there is a scant number of patients (5) who are I/I carriers (8.3% of all patients), nevertheless 3 (60%) have mild dementia, whereas none have severe symptoms. Conversely, patients in the top scoring MMSE category (mild dementia) are more likely to be I/D carriers (67%) followed by I/I carriers (25%). However, only 1 patient is a D/D carrier suffering with mild dementia. The effects are significant: The odds are 4 times larger for suffering severe versus moderate symptoms (*p* = .04) and 37 times larger for suffering severe versus mild symptoms (*p* < 10^−3^) for AD patient D/D carriers as compared to non-D/D carriers. The analysis of the alleles reveals a similar trend. Patient carriers of the D allele are predominantly sufferers of severe dementia (53%), 35% have moderate dementia, whereas 12% have mild dementia. Conversely, 88% of those with severe dementia are D carriers. Overall, the odds are 3.6 times higher for scoring in the lowest MMSE category (severe dementia) as compared to the moderate category (*p* = .02) for D carriers. The odds are 11 times higher for scoring in the lowest MMSE score category as compared to the highest score category, i.e., those with mild dementia (*p* < 10^−3^). Given the patients in this study, with an average age at AD diagnosis of 69 years and evaluated at an average age of 75 years, those suffering severe dementia are statistically more likely to be D/D carriers (or D allele carriers) and, conversely, D/D (or D carriers) are statistically more likely to suffer severe dementia.

**Table 3:** MMSE severity by ACE I/D genotypes and alleles in Alzheimer’s patients

ACE I/D genotype and alleles *n* (%) *N* = 60	MMSE ranges *n*% (*C*, *R*, *T*)			Severe vs. moderate Severe vs. mild	
	0–9 (severe) *N* = 26	10–18 (moderate) *N* = 22	19–23 (mild) *N* = 12	OR [CI 95%]	*p* values
II 5 (8)	0 (0, 0, 0)	2 (9, 40, 3)	3 (25, 60, 5)	– 96 [3.2–2800] (*)	.13 *.002*
ID 24 (40)	6 (23, 25, 10)	10 (45, 42, 17)	8 (67, 33, 13)	3.3 [.94–12] 27 [2.8–260]	.07 *<* *10* ^*−3*^
II + ID 29 (48)	6 (23, 21, 10)	12 (55, 41, 20)	11 (92, 38, 18)	4 [1.2–14] 37 [3.9–340	.04 *<* *10* ^*−3*^
DD 31 (52)	20 (77, 65, 33)	10 (45, 32, 17)	1 (8, 3, 2)	– –	– –
I 34 (28)	6 (12, 18, 5)	14 (32, 41, 12)	14 (58, 41, 12)	3.6 [1.2–10] 11 [3.3–35]	*.02* *<* *10* ^*−3*^
D 86 (72)	46 (88, 53, 38)	30 (68, 35, 25)	10 (42, 12, 8)	– –	

DD versus II, ID, and I* genotypes; Statistics calculated using Fisher’s exact test; (*) Haldane’s correction used; *CI* confidence interval, *% (C, R, T)* column %, row %, % of total (all % rounded to nearest whole percentage point), *OR* odds ratio, *p value* probability value

Significant *p* values are in italics

## Discussion

This study analyzed the frequencies of the ACE I/D polymorphism in AD patients in comparison with a control group. The D/D genotype (*p* = .008, OR = 2.32) and D allele (*p* = .003, OR = 2.0) were found to be risk factors for AD. The inheritance of the homozygous I/I genotype was marginally associated (*p* = .06) with a reduced risk for AD and the ACE I/D heterozygotes showed no statistical differences in frequencies between subjects and controls (*p* = .24).

Moreover, given the particular demographics of our patient group, D/D carriers were more likely to score in the lowest category of MMSE scores (severe dementia) as compared with I/* carriers. Additionally, there were only 8 I/I carriers of whom none suffered from severe dementia as measured by the MMSE.

The ACE insertion–deletion polymorphism has been frequently studied as a genetic risk factor for AD but the results detailing the relationship between ACE indel and AD are inconsistent. Conflicting results prompt questions as to whether the polymorphism is a risk factor at all and, when answered in the affirmative, lead to further disagreements as to which genotype(s) is (are) the responsible risk factor(s).

Several studies have failed to find any association implicating the I/D polymorphism as a risk factor for AD [15–18]. In fact, a recently conducted meta-analysis suggested that the ACE I/D polymorphism is unlikely to be a major determining factor in the development of AD [10].

In contrast, however, other studies have determined the ACE indel polymorphism to be a risk factor for AD but they disagree as to which allele and genotype(s) are the responsible factor(s).  For example, several studies have revealed associations of the I allele (and/or I/D genotype) with an increased risk of AD and, conversely, the D/D genotype with a lower risk of AD [19–26]; whereas, there are studies in diametric opposition that implicate the ACE D allele as a risk factor [27–30].

Here, our findings demonstrated that the ACE D allele frequency was significantly increased in the AD group as compared to controls. In particular, our results are in agreement with similar research conducted with a Tunisian population where the authors reported an increased risk of AD in D/D genotype carriers [31].

The lack of coherency among ACE-AD investigations may be explained by the differing genetic backgrounds among ethnicities or, in some cases, possibly intra-population heterogeneities. A meta-analysis that included three ethnic groups (North European, South Caucasian, and East Asian subjects) addressing the relationship between the ACE indel polymorphism and AD has shown ethnic-dependent results. Although all groups revealed that the D homozygotes were at reduced risk of AD, there was no overlap whatsoever in allelic frequencies among the three groups. Heterozygotes were at increased risk in North Europeans, whereas I homozygotes were at higher risk in East Asians [9]. There is evidence, however, that ethnicity alone, in a genetically heterogeneous population, may not prove to be sufficient to explain conflicting results. For example, a study from Japan found that the ACE I/I genotype is associated with an increased prevalence of AD, only to be contradicted by other Japanese studies [22, 32].

The present study with Tunisian subjects benefits from the determination that populations of the Maghreb show a substantial degree of genetic homogeneity, regardless of culture and geography [33, 34]. This is corroborated, in part, by the only other ACE-AD study to date with Tunisian subjects that also report commensurate genotype frequencies with those subjects of the present study [31].

The mechanism whereby the ACE I/D polymorphism could contribute to the development of AD pathology remains contentious. In general, explanations can first be parsed into two main categories that depend on the dual character of ACE.

Serum ACE levels have been found to be associated with ACE I/D, with the highest serum ACE levels in subjects with ACE genotype D/D and the lowest serum ACE levels in subjects with genotype I/I [5, 18, 35, 36]. ACE promotes the formation of angiotensin II from angiotensin I and inactivates the vasodilator bradykinin; the net result is vasopressin activity [37, 38]. Such a perturbation in vasoconstriction for the regulation of blood pressure may likely influence normal cell function in many different tissues, such as in vascular endothelia which, in turn, may cause neural cell degeneration and contribute to the development of dementia. Increasingly, the actions of ACE and, therefore, angiotensin II within the central nervous system are of increasing interest in the context of Alzheimer’s disease [4, 35, 39].

Another proposed mechanism for AD development is explained via the amyloid hypothesis which suggests that the deposition of beta-amyloid (Aβ) is a primary event in the pathological cascade for AD. Research indicates that ACE cleaves amyloid-β (Aβ) and, therefore, inhibits amyloid-β peptide aggregation and thus plaque formation [8, 40, 41].

The alteration of activity of this enzyme in the central nervous system (CNS) may have an impact upon the Aβ accumulation thereby resulting in senile plaque which is central to the pathogenesis of Alzheimer’s disease. The research implicating the I allele as a risk factor and the D allele as protective in AD [15, 17–22, 35] is consistent with this hypothesis given that ACE activity is highest in D homozygotes [5, 36].

There have been proposed several hypotheses that attempt to reconcile the trichotomous associations of the ACE I/D polymorphism with AD (no association versus D versus I as risk factors) given the dual activities of ACE (cleavage of Aβ versus possible vascular dysfunction via endothelia damage). The proposals include a theory of homeostasis disruption whereby increased levels of ACE mediate short-term Aβ clearance but that up-regulation of Ang II in turn causes hypertension, damage to the blood–brain barrier, reduced cerebral blood flow results in de novo Aβ deposition, inflammation, regional white matter volume changes, and reduced cholinergic activity [35, 42–44]. Another explanation advanced to explain the findings of contrary associations between ACE I/D and AD is that this polymorphism is in varying linkage disequilibrium, depending on ethnic group, to other single nucleotide polymorphisms (e.g., rs4343, rs4335, and rs4291) that have stronger associations with AD [31, 38, 43, 45, 46]. Yet other research, while at times finding ACE I/D-AD associations and in other instances finding no independent ACE I/D-AD association, has nevertheless found associations or more pronounced effects when gene–gene interactions (e.g., ACE I/D-apolipoprotein E) were considered [18, 35, 46]. This suggests that the ACE I/D polymorphism may be, at a minimum, an effect modifier in the etiology of AD.

The present research also found an association of ACE D/D carriers with the lowest MMSE scores as compared with I carriers. Other researchers have also investigated this association with varying results. Yip et al. did not find any association between ACE I/D and AD [47]. Similarly, a Danish study failed to find an association between ACE genotypes and cognitive decline [48]. However, Richard et al. found that DD carriers had the lowest cognitive scores and that cognitive decline was more prevalent in these subjects when compared with I/D subjects as a reference class (Moreover, they report that the combined effect of the presence of at least one APOE ε4 allele and D homozygosity was a risk factor for cognitive decline) [49]. A Greek study reported that subjects who were double homozygous [ACE DD and TNF GG (tumor necrosis factor)] presented with significantly decreased MMSE scores as compared to other double genotypes [50]. Similarly, a study involving an African–Caribbean population found an association between increased age and cognitive decline was significantly stronger in people with the ACE DD genotype [51]. In contrast, however, a study by Chou et al. found that AD patients, homozygous for the I allele, presented with a more rapid AD deterioration than did those who had other ACE genotypes as measured by the MMSE [52].

It is notable that the debate between the association of ACE I/D and AD and the corresponding debate between the opposing natures of ACE (and Ang II) on the central nervous system is mirrored in the current debate over the clinical use of ACE inhibitors (ACE-Is) in AD beyond their role as a treatment for hypertension. While some research indicates that the use of ACE-Is (or angiotensin receptor blockers) in older adults with AD is associated with a slower rate of cognitive decline [42, 53–55], other research cautions in the use of ACE inhibitors as they may interfere with Aβ clearance [40, 56, 57].

Currently, only the APOE ε4 allele is widely accepted as a risk factor for AD. However, approximately 42% of persons with late-onset AD are not APOE ε4 carriers. Thus, the absence of the APOE ε4 allele does not rule out an AD diagnosis [58]. Research has also found ACE to be over-expressed in the hippocampus, frontal cortex, and caudate nucleus of patients with AD [55]. Consequently, study of the ACE I/D polymorphism has become a subject of interest in the etiology of AD.

The present research would have benefited from a larger sample size to increase the power of the results. A larger sample would also allow stratification by AD subtypes and allow a more detailed study of the association of genotypes with MMSE scores regressed upon age, sex, and duration of disease. Future research directions should include investigations of variants in linkage disequilibrium with ACE I/D, examining associations between ACE I/D genotypes and ACE levels (peripheral and in the central nervous system), study of gene–gene interactions (e.g., ACE I/D with APOE), and longitudinal, genotype association studies of MMSE score progression and the study of disease evolution among ACE inhibitor cohorts.

## Conclusions

In summary, our study shows an association of the ACE gene I/D polymorphism with AD in a Tunisian population. The D/D genotype confers significant susceptibility for AD and is associated with lower MMSE scores among AD patients as compared to other genotypes in the Tunisian population. These preliminary exploratory results should be confirmed in a larger study and further work is required to discern among possible differing findings in diverse populations.

## Abbreviations

ACE: angiotensin-converting enzyme
Ach: acetylcholine
Ang II: angiotensin II
AD: Alzheimer’s disease
Aβ: amyloid-β
APOE: apolipoprotein E
CI: confidence intervals
I/D: insertion/deletion
MMSE: Mini-Mental State Examination
MRI: magnetic resonance imaging
NINCDS–ADRDA: National Institute of Neurological and Communicative Diseases and Stroke/Alzheimer’s Disease and Related Disorders Association
OR: odds ratio
